# Corona-Krise: (Wirtschafts-)politische Perspektiven: Die Reflexe aus der Finanzkrise sind nicht genug!

**DOI:** 10.1007/s10273-020-2630-6

**Published:** 2020-04-22

**Authors:** Jan Philipp Fritsche, Patrick Christian Harms

**Affiliations:** 1 Makroökonomie, DIW Berlin, Mohrenstraße 58, 10117 Berlin, Deutschland; 2 Wirtschafts- und Sozialwissenschaften, Universität Hamburg, Welckerstr. 8, 20354 Hamburg, Deutschland

**Keywords:** E50, E60, I10

## Abstract

Die Deutsche Gesellschaft für Epidemiologie warnte, dass die Zahl der COVID-19-Fälle schon bald die Kapazität des Gesundheitssystems übersteigen könnte. In Deutschland und weltweit droht eine schwere Rezession aufgrund eines externen Schocks. Mögliche Szenarien zur Bekämpfung der Krise zeigen, dass drastische Maßnahmen zur Eindämmung des Virus die beste Lösung für die Krise bleiben. Die Entwicklungen unterscheiden sich fundamental von denen der globalen Finanzkrise 2008. Während der Euroraum in Bezug auf Liquiditäts- und Finanzhilfen für Unternehmen und Staaten bereits besser gewappnet ist als noch 2009, fehlt die Praxis in der Zusammenarbeit von Gesundheitsforschung und Wirtschaftspolitik. Maßnahmen auf der Nachfrageseite werden die Epidemie kurzfristig nicht heilen. Eine koordinierte Finanzpolitik und gemeinsame europäische Anleihen können vor allem gefährdete Staaten schützen.

## Abstract (en)

The explosion of Covid-19 cases is looming in Germany. The German Society for Epidemiology has warned that the number of cases could soon overshoot the capacity of the healthcare system. This may be true even if Germany follows the ‘flatten-the-curve’- approach to reduce infection rates. A suppression of the virus remains the best solution for the crisis. Supply will suffer as long the virus persists. Until then, demand side measures will not cure the epidemic. Coordinated measures for business that ensure compliance and European debt instruments may be part of a strategy to solve the crisis.

Die weitere Verbreitung des Virus in China scheint erst einmal gestoppt (Cyranoski, 2020), während der Rest der Welt in eine tiefe Rezession zu stürzen droht. Bislang haben sich die wenigsten Staaten dazu entschlossen, der fortschreitenden Ausbreitung so entschieden zu begegnen, wie China es gewagt hat. Der niedrige Ölpreis lässt zweifeln, ob die Produktion in China tatsächlich schnell wieder anziehen wird. Unklar ist auch, ob es überhaupt gelingen kann, das Virus nach Abschluss der Maßnahmen und bei einer Rückkehr zur Normalität nachhaltig zurückzudrängen.

Sollte die Verbreitung und Letalität des Virus sich so entwickeln, wie bisher prognostiziert, drohen deutliche Einschnitte in unser Wirtschaftsleben und Miteinander (Deutsche Gesellschaft für Epidemiologie, 2020). Ein Risikoszenario für die politische Reaktion ist, dass diese zwar zunächst die Wirtschaft lähmt, aber nicht beherzt genug gegen die Verbreitung vorgeht. Ohne Frage erleichtern es die aus der Finanzkrise erlernten Reflexe, rasch Liquidität zur Verfügung zu stellen, den Unternehmen für eine gewisse Zeit zu überleben. Fraglich ist aber, ob die Bevölkerung von alleine verantwortungsvoll gegen die Verbreitung vorgeht.

Während die Trump-Administration in den USA sich mit der Aktivierung ihres Defense Production Act umfassende Befugnisse gesichert hat, auf die Produktion und die Steuerung der Unternehmen einzuwirken, bleiben solche Bemühungen im Euroraum bisher aus (The White House, 2020). Europa braucht schnell eine klare Strategie zur Bekämpfung der drohenden Herausforderungen, damit die ganze Bevölkerung daran mitwirken kann und von der Unsicherheit befreit wird. Eine transparente Kommunikation hat jetzt oberste Priorität. Brachliegende Ressourcen sollten so weit wie möglich genutzt werden – um die medizinische Versorgung sicherzustellen oder zu verbessern. Schließlich muss die Krise als europäisches Problem begriffen und bewältigt werden. Leidet ein Staat, leiden alle.

### Hergang der Krise

Der COVID-19-Schock ist zunächst ein rapides und starkes Absinken des gesamtwirtschaftlichen Angebots. Der Großteil der früh ergriffenen Maßnahmen (Absagen von Veranstaltungen, Quarantäne, Isolation etc.) führt unmittelbar zu einem Rückgang des Arbeitsvolumens und damit zu einem Einbruch bei der Produktion von Waren und Dienstleistungen und schließlich auch der Einkommen. Derzeit ist davon auszugehen, dass sich diese Maßnahmen über mehrere Quartale erstrecken, wodurch eine technische Rezession unvermeidlich erscheint (Michelsen et al., 2020). Grundsätzlich dürfte die eigentliche Ursache dieser Angebotseinbrüche wieder verschwinden, sobald die Verbreitung des Virus gestoppt und die Maßnahmen, die dies erzwingen sollen, gelockert wurden. Allerdings bedeutet die Stilllegung des gesamtwirtschaftlichen Angebots für viele Unternehmer erhebliche Umsatz- und Gewinnrückgänge sowie Lohn- und Einkommenseinbußen für Arbeitnehmer.

Da nicht nur die inländische Wirtschaft von dem Virus betroffen ist, sondern es sich um ein globales Phänomen handelt, sind auch die internationalen Lieferketten beeinträchtigt, wodurch ein erneutes Absinken des globalen Handelsvolumens droht. Durch die Lieferengpässe bei Vor- und Zwischengütern werden die Produktionsausfälle noch einmal verschärft. Hiervon sind international eng vernetzte Volkswirtschaften wie Deutschland besonders stark betroffen.

Die Finanzmärkte reagierten bereits mit Kurseinbrüchen, die an Panikverkäufe erinnern. Risikoreiche Anlageformen werden derzeit gemieden und sichere Anlageformen wie Gold- und Bundesanleihen erlebten deutliche Kursgewinne. Grundsätzlich bedeutet diese Bewegung einen Vermögensverlust bei privaten und institutionellen Investoren, die an den Aktienmärkten investiert gewesen sind, und verschlechtert gleichzeitig die Finanzierungsbedingungen der betroffenen Unternehmen. Bei Unternehmen und Branchen, die von bestimmten Maßnahmen besonders betroffen sind (Airlines, Hotels, Catering, Restaurants), könnte auch ein vergleichsweise kurz andauernder Krisenverlauf bereits zu Insolvenzen führen. Die Unsicherheit an den Finanzmärkten ist derzeit ebenso groß wie die Hoffnung auf politische Maßnahmen zur Stabilisierung.

Auch ohne stützende fiskalpolitische Maßnahmen führt die Rezession zu deutlichen Ausgabensteigerungen und Steuerausfällen bei den Staaten, wodurch die Staatsverschuldung massiv ansteigen wird. Alle Fiskalregeln – wie Fiskalpakt und Schuldenbremse – sehen für derartige Krisen Ausnahmen vor und lassen deutlich höhere Niveaus der Neuverschuldung zu. In besonders verwundbaren und stark betroffenen Staaten – wie Italien und Spanien – steigt daher das Risiko für Zahlungsausfälle der öffentlichen Hand, die jetzt schon mit höheren Renditeaufschlägen etwa gegenüber Bundesanleihen leben müssen. Nichtsdestotrotz erscheint die Schuldentragfähigkeit derzeit auch aufgrund des weiter andauernden Ankaufprogramms von Staatsanleihen weitestgehend gegeben und der Euroraum ist auf derartige Ausfälle durch den Europäischen Stabilitätsmechanismus (ESM) und die Möglichkeit der geldpolitischen Outright-Geschäfte besser vorbereitet als in der Vergangenheit.

All diese Mechanismen sind von temporärer Natur und die Lage wird sich mehr oder weniger sofort wieder entspannen, wenn Produktion und Dienstleistungen wieder in größerem Umfang bereitgestellt werden. Ob die Situation sich daher auf eine tiefgreifende Krise des Finanzsystems auswächst, ist gegenwärtig nicht absehbar. Aus diesem Grund ist die entscheidende Frage derzeit nicht, ob mit einem Einbruch oder einer Rezession zu rechnen ist – diese gilt als sicher –, sondern wie lange der gesellschaftliche und ökonomische Ausnahmezustand anhält. Alle beschriebenen Probleme verschlimmern sich mit der Dauer der Krise und es dürften mit anhaltend niedrigem Angebot diverse weitere Probleme hinzukommen. Für eine makroökonomische Einschätzung ist daher der Verlauf der Virusverbreitung und die Durchführung entsprechender Maßnahmen zur Bekämpfung von zentraler Bedeutung.

**Jan Philipp Fritsche**, M. Sc., ist Mitarbeiter und Doktorand am DIW Berlin.

**Patrick Christian Harms**, M. Sc., ist wissenschaftlicher Mitarbeiter an der Fakultät für Wirtschafts- und Sozialwissenschaften der Universität Hamburg.

## Möglicher Verlauf

Ein großer Unterschied zwischen den Krisen von 2008 und heute ist die fehlende Koordination zwischen den Disziplinen. Während die Krise 2008 ihren Ausgang an den Finanzmärkten nahm und damit nah am Tagesgeschäft der Wirtschaftswissenschaften, ist die Wissenslücke heute größer. So kommt es zu einem Ping Pong von Argumenten zwischen Wirtschaftswissenschaften und Epidemiologie statt einer koordinierten Zusammenarbeit, wobei wertvolle Zeit verschwendet wird. Auch das Verhalten der Politik scheint durch diese Verständnislücke geprägt zu sein. Während in China sogar der Bau von Krankenhäusern veranlasst wurde, wird in der restlichen Welt weitgehend mit finanziellen Hilfspaketen jongliert, die nicht notwendigerweise direkt am Problem ansetzen. Ressourcen von Unternehmen liegen jetzt ungenutzt brach. Sie für die Bekämpfung der Pandemie zu nutzen, könnte zur Bekämpfung der Pandemie beitragen und wäre langfristig auch für die Wirtschaft von Vorteil. Sogar ein Strukturwandel zu nachhaltigeren, regionaleren, resilienteren und digitaleren Wertschöpfungsketten, könnte durch das Tacet vereinfacht werden, wo er politisch gewollt ist.

Aus vorherigen Krisen haben wir gelernt, dass Verständlichkeit, Transparenz und Schnelligkeit wichtige Erfolgsfaktoren sind. Eine gelungene Koordination der Wissenschaften müsste jetzt schnell eine konsistente Strategie formulieren, um bestimmte Kernfragen zu beantworten. Nur so können Unternehmen und die Bevölkerung sich darauf einstellen und im Idealfall an ihr mitwirken. Dazu gehören die Fragen: Für wie lange und wie stark sollen der soziale Kontakt und das wirtschaftliche Leben eingeschränkt werden? Welche Kapazitäten sollen in Krankenhäusern geschaffen werden? Wie viele Personen sollen oder dürfen sich pro Tag infizie-

Abbildung 1

**Skizzen möglicher Szenarien**

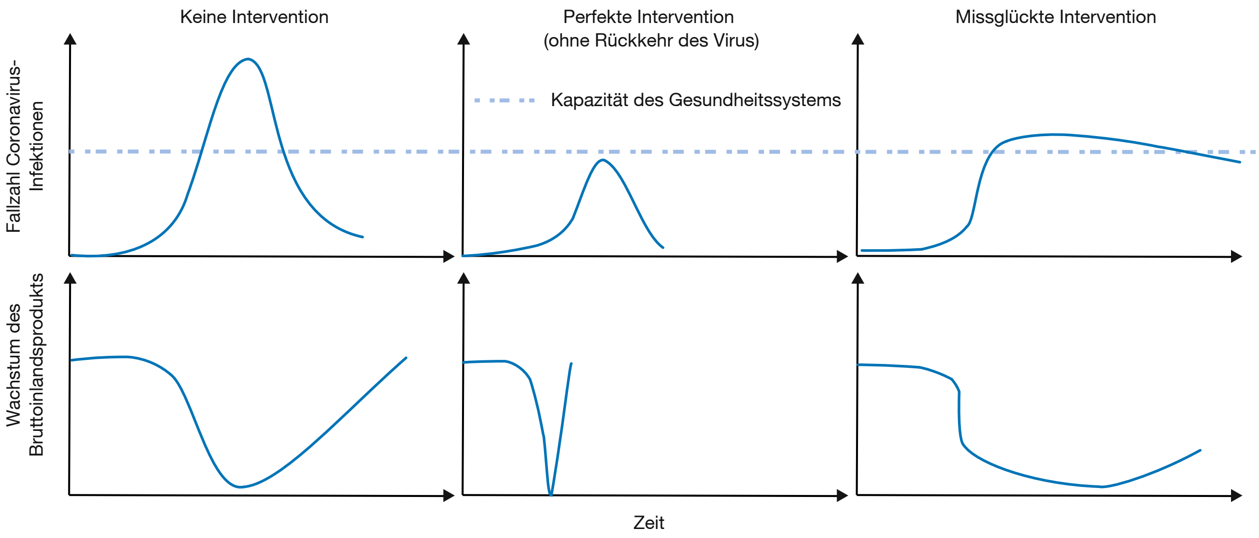

Quelle: eigene Grafik.

ren? Bisher änderte sich die Intensität der Maßnahmen rasch und die Bevölkerung reagierte teils trotzig auf die Empfehlung soziale Kontakte zu meiden. Wohl auch aus dem Grund, dass bisher nicht klar ist, für wie lange welches Ziel verfolgt werden soll.

Für eine Herdenimmunität müssten sich etwa 60 % der Bevölkerung durch überstandene Infektion immunisieren. Wenn dieser Wert (analog zur britischen Strategie, die sich exakt einen Tag hielt bis sie wieder verworfen wurde) noch vor der Erkältungswelle im Herbst erreicht werden soll, müssten wir pro Tag über 200.000 neue Infektionen verzeichnen. Würden wir die derzeitigen Infektionsraten linearisieren, würde es mehrere Jahre dauern, bis die Herdenimmunität erreicht ist. Soll die Ausbreitung des Virus durch tiefgreifende Maßnahmen gestoppt werden und falls ja, wie wird der Wiederausbruch mit erneutem Stillstand verhindert? Derzeit kann die Haltung der Politik nur so gedeutet werden, als würde durch eine etwas langsamere Ausbreitung des Virus versucht, ein wenig Zeit zu gewinnen. Eine schleichende Ausbreitung des Virus mit einer gesenkten Produktion für einen längeren Zeitraum könnte aber auch schlimmere Folgen haben als ein kurzfristiger tiefer Einschnitt.

Die Abbildung 1 zeigt extreme Verläufe von Fallzahlen und wirtschaftlicher Entwicklung. Ganz links, ein Szenario mit ungebremster Ausbreitung des Virus über die Kapazitätsgrenze des Gesundheitssystems hinaus und anschließendem wirtschaftlichen Kollaps. In der Mitte ein Traumszenario für Wirtschaft und Bevölkerung: Die Ausbreitung wird durch einen fast vollständigen Stillstand des Wirtschaftens gestoppt und das Virus kehrt auch nach dem Wiederanlauf nicht zurück. Ganz rechts die schleichende Katastrophe, bei der durch zu zaghafte Maßnahmen die Wirtschaft zwar geschwächt wird, die kritische Ausbreitung des Virus aber nicht verhindert wird und schließlich langfristig den bereits geschwächten Wirtschaftskreislauf trifft. Diese fiktiven Extremszenarien sollen im Wesentlichen die Planungsabhägigkeiten illustrieren, die zur Beantwortung der strategischen Fragen zu bedenken sind. Mit etwas Glück sind all diese Gedanken, sobald es Sommer wird, obsolet – an dieser Stelle fehlt uns die epidemiologische Expertise.

## Was unterscheidet die Krise von 2008?

Schon die Namensgebung von Corona-Krise und Finanzmarkt-Krise zeigt, dass sich die beiden ihren Ursachen nach unterscheiden. Was die Auswirkungen betrifft ist zum gegenwärtigen Zeitpunkt allerdings unklar, inwiefern es Parallelen oder Unterschiede geben wird. Folgt man den drei Fällen in dem Schaubild, so dürfte nur der Fall in der Mitte, bei dem die Ausbreitung des Virus schnell gestoppt und sich die Krise auf wenige Monate begrenzt, mildere Auswirkungen als die Krise von 2008 mit sich bringen. In diesem Fall wäre eine Überbrückung der akuten Krise der Schlüssel zur Lösung des Problems und die Konjunktur dürfte ähnlich beeindruckend zurückkommen, wie sie durch das Virus in die Knie gezwungen wurde. In den beiden anderen Fällen, wo von einer länger anhaltenden Schwächephase ausgegangen wird, wären die makroökonomischen Konsequenzen schwerwiegender.

Der negative Angebotsschock ist nur für sehr begrenzte Zeit für die Gesamtwirtschaft verträglich. Je länger diese Krise dauert, desto höher ist die Wahrscheinlichkeit für massenhafte Firmeninsolvenzen, Einkommens- und Vermögensverluste, Kreditausfälle und eine deutliche Eintrübung der Staatsfinanzen. Insbesondere der letzte Punkt ist aus zwei Gründen wichtig: Zu Beginn der Krise von 2008 hatten viele Regierungen es mit deutlich niedrigeren Schuldenstandsquoten zu tun als heute, sodass der fiskalische Spielraum in einigen Fällen stärker begrenzt sein dürfte als damals. Darüber hinaus waren die Zentralbanken weltweit damals noch in der Lage einen Teil des Einbruchs durch Zinssenkungen abzufedern. Weil die kurzfristigen Zinsen im Euroraum kaum weiter sinken können, müssen weniger erprobte Instrumente zur Dämpfung eingesetzt werden. Darüber hinaus erscheint die Koordinierungsfähigkeit der Regierungen durch die nationalpopulistischen Tendenzen der letzten Jahre im Vergleich zu 2008 stark eingeschränkt zu sein, sodass alle ergriffenen Maßnahmen aufgrund fehlender Koordinierung weniger effektiv zu sein drohen.

In diesem Zusammenhang ist das Szenario „missglückte Intervention“ die größte Gefahr, da die Krise in diesem Fall am längsten andauert. Die beschriebenen ökonomischen Probleme würden hier am stärksten zutage treten und es muss mit einer alleine aufgrund des Virus mehrjährigen Schwächephase gerechnet werden, die das Potenzial hat, die Weltwirtschaft mittel- bis langfristig zu schwächen. Vor allem bei der Suppressionsstrategie, die hier aus gesundheitlicher und ökonomischer Perspektive als das Ideal hervorgehoben wird, muss es einen weiteren Plan für die Zeit nach der ersten Eindämmung geben. Unter dem Titel „The Hammer and the Dance“ ist eine solche Strategie mittlerweile bekannt geworden. Sie zielt darauf ab, zunächst mit drastischen Maßnahmen die Ausbreitung einzudämmen (Hammer) und in der darauffolgenden Zeit mittelfristig die Maßnahmen schrittweise zu lockern und wenn nötig wieder zu straffen (Dance) (Pueyo, 2020). Wie die Süddeutsche Zeitung berichtet, hat diese Strategie es mittlerweile in ein nicht veröffentlichtes Strategiepapier der Bundesregierung geschafft (Richter, Mascolo and Charisius, 2020). Eine wissenschaftliche Schwachstelle dieses Szenarios ist jedoch, dass es eine wesentlich kürzere Aufrechterhaltung der sozialen Distanzierung annimmt, als aus wissenschaftlicher Sicht gerechtfertigt wäre (DGEpi, 2020; Ferguson et al., 2020).

## (Wirtschafts-)politische Antworten

Die Tendenz, gesundheitliche Probleme nicht primär politisch zu adressieren, muss jetzt gebrochen werden. Als 1842 das erste Kanalisationssystem Deutschlands in Hamburg gebaut wurde, geschah dies als Reaktion auf einen gigantischen Brand, der eine Trinkwasserknappheit zur Folge hatte. Seit der neoklassischen Wende werden moderne Gesundheitsprobleme tendenziell eher dem Individuum oder den Unternehmen überlassen. Möglicherweise muss diese Haltung nun überwunden werden. Eine Devise könnte lauten: Gebt den Menschen klare Anweisungen und besser etwas zu tun, das gegen den Virus hilft, als nur Geld. So kann auch die Wirtschaft im Notbetrieb erhalten werden.

Die Reaktion der Geldpolitik fällt weltweit relativ unterstützend aus. In China, wo bereits Straßenmusiker mit WeChat bezahlt werden können, sticht besonders eine Maßnahme der Zentralbank heraus. Zwar wurden auch der Geldmarkt und die Wirtschaft gestützt, aber die innovativste Maßnahme war wohl, das zirkulierende Bargeld zu desinfizieren und zu versiegeln und so bis zu 14 Tage aufzubewahren. Gleichzeitig wurde in der am stärksten betroffenen Provinz Hubei nicht kontaminiertes Bargeld in Umlauf gebracht. Die Zentralbank von Hongkong reagierte ebenso ungewöhnlich. Sie plant, einmalig 10.000 Hongkong-Dollar (ca. 1.180 Euro) an alle Einwohner als Direkthilfe auszuzahlen, um der Krise zu begegnen.

Am weltweit deutlichsten reagierte bisher die US-Notenbank, die nach einer anfänglichen Zinssenkung um 0,5 Prozentpunkte Anfang März, direkt eine weitere drastische Reduktion von einem Prozentpunkt folgen ließ. Die Zinsen liegen in den USA damit jetzt wieder auf dem Krisenniveau von 0 % bis 0,25 %. In Europa fiel die geldpolitische Reaktion zunächst geringer aus, auch wenn das Anleihenkaufprogramm deutlich ausgeweitet wurde. In der Nacht vom 18. auf den 19. März 2020 verabschiedete die EZB ein historisches und 750 Mrd. Euro schweres Notfallprogramm (European Central Bank, 2020). Es lässt Abweichungen vom Kapitalschlüssel zu. Darüber hinaus wurde der Sicherheitsrahmen ausgeweitet. Ebenso stellte die Notenbank in ihrer Pressemitteilung klar, dass sie bereit ist alle selbst auferlegten Beschränkungen zu beseitigen, sofern sie die Wirkung der Maßnahmen der EZB in einem der Mitgliedsländer behindern. Sollte sich die Krise verschärfen, hat die EZB jetzt noch deutlichen Spielraum: von einer weiteren Lockerung bis zum Helikoptergeld (Fritsche und Steininger, 2019). Auch für den absoluten Notfall stehen die Mittel bereits bereit: Sollte ein Staat in Zahlungsschwierigkeiten geraten, stehen der ESM und an ihn gekoppelt die geldpolitischen Outright-Geschäfte (OMT) zur Verfügung. An dieser Stelle sind wir also weiter als zur Zeit der Eurokrise.

## Fiskalpolitik

Die Bundesregierung hat neben den gesundheitspolitischen Maßnahmen bereits auf verschiedene Arten auf die ökonomische Krise reagiert. Dazu zählen eine Vereinfachung und Ausweitung des konjunkturellen Kurzarbeitergeldes und eine Garantie für von den Geschäftsbanken an Unternehmen gewährte Überbrückungskredite über die Kreditanstalt für Wiederaufbau. Darüber hinaus haben Bundesfinanz- und

Wirtschaftsminister gemeinsam eine Garantie für alle jetzt in der Corona-Krise vergebenen Kredite ausgesprochen und die Möglichkeit von Steuerstundungen angekündigt. Damit kann davon ausgegangen werden, dass – so lange die von der Geldpolitik veranlassten Maßnahmen ihr Ziel erreichen und die Liquiditätssituation im Finanzsystem stabil halten – kein Unternehmen alleine aufgrund unzureichender Liquidität den Betrieb einstellen muss. Allerdings sind die Umsatz- und Gewinneinbußen teilweise so groß, dass insbesondere in kleineren und mittleren Unternehmen die dünne Kapitaldecke schnell aufgezehrt werden könnte. Derzeit werden daher diverse weitere Ideen diskutiert, die insbesondere Kleinunternehmern und Selbstständigen sowie besonders stark betroffenen Branchen durch die Krise helfen sollen.

Dieser Katalog an Maßnahmen folgt grundsätzlich der Idee einer kurzfristigen Krise, die nach dem Ende der gesundheitlichen Krise beendet ist, wenn die Maßnahmen zur Eindämmung gelockert werden. Es ist derzeit nur indirekt einzusehen, dass weiterreichende fiskalpolitische Expansionen zur Stimulierung der Nachfrage während der akuten Corona-Krise hilfreich sind. Der Angebotsschock und damit der Einbruch der Produktion wird in jedem Fall so lange bestand haben, wie die Ausbreitung des Virus nicht gestoppt ist und die Maßnahmen zur Eindämmung andauern. Gleichzeitig gibt es derzeit keinen Grund anzunehmen, dass die einzelnen Nachfragekomponenten selbst im Falle der idealtypischen, schnellen Beendigung der Krise sofort und vollständig auf ihr altes Niveau ansteigen. Beinahe alle Haushalte, die mit Kurzarbeit, Arbeitslosengeld I, Umsatzeinbußen bei unternehmerischer Tätigkeit oder Vermögensverlusten konfrontiert werden, nehmen Einkommensverluste hin, die die Konsumnachfrage schwächen können. Die deutsche Wirtschaft dürfte durch ihre internationale Verflechtung besonders stark von dem weiteren Absinken des Welthandelsvolumens betroffen sein und die Investitionen werden aufgrund der erwartbar erhöhten Unsicherheit verhalten bleiben. Daher ist spätestens nach Ende der akuten Krise eine fiskalpolitische Expansion über die automatischen Stabilisatoren hinaus angezeigt.[1]

Im Euroraum sind mit Deutschland, Frankreich, Italien und Spanien alle größeren Volkswirtschaften massiv von der Ausbreitung des Virus betroffen und alle haben mit weitreichenden und einschränkenden Maßnahmen reagiert. In Spanien und insbesondere in Italien dürften die fiskalischen Belastungen die Regierungen vor große Herausforderungen stellen. Bei einer andauernd niedrigen ökonomischen Aktivität könnte die italienische Regierung aufgrund der hohen Staatsverschuldung von etwa 135 % mit dem Ausgabenanstieg und Einnahmeneinbruch überfordert sein und Probleme bei der Refinanzierung bekommen. Je nach Verlauf der Krise ist dies auch für andere schwächer aufgestellte Volkswirtschaften zu erwarten. Eine solche Entwicklung mit drastisch auseinandergehenden Zinsdifferenzen zwischen den Volkswirtschaften, gestiegener Unsicherheit um die Zahlungsfähigkeit einzelner Staaten und Kapitalflucht gilt es zu vermeiden. Daher sind die neuesten Diskussionen um gemeinsame Anleihen der Staaten des Euroraums zu begrüßen, um nicht wieder der EZB eine eigentlich fiskalpolitische Aufgabe aufzubürden. Diese könnte erst nachdem einzelne Regierungen einen Antrag auf Finanzierung aus dem ESM gestellt haben, im Rahmen des OMT-Programms gezielt Anleihen einzelner Länder kaufen.[2]

Ähnlich wie die Hongkonger Zentralbank kündigte auch die amerikanische Trump-Administration bereits Helikoptergeld von zwei Schecks je 1.200 US-$ für die Bevölkerung an (Mascaro, Taylor und Lemire, 2020). Dazu sollen starke Steuererleichterungen und Notfallkredite für Unternehmen kommen. In Deutschland wird auf Bundesebene ebenso über Helikoptergeld diskutiert, während der Berliner Bürgermeister Müller bereits 5.000 Euro für alle Solo-Selbstständigen als unbürokratische Direkthilfe angekündigt hat. Falls Helikoptergeld im größeren Stil in Europa ausgeschüttet werden sollte, wird es wahrscheinlich eher eine Maßnahme der EZB werden oder aus einem europäischen Budget kommen. Obwohl der Begriff mittlerweile Synonym für bürokratiefreie Transferzahlungen an die Bevölkerung benutzt wird, ist dabei zu bedenken: Während Helikoptergeld durch den Staat den Haushalt belastet, führt der direkte Transfer durch die Zentralbank niemals zu einem Anstieg der Schulden.[3]

Die Vermögensmärkte haben sich trotz der Maßnahmen bisher nicht erholt, die Arbeitslosenzahlen der USA drohen auf das Niveau der Finanzkrise zu schnellen und die Bevölkerung ist weiterhin uneinig über die Sinnhaftigkeit der Maßnahmen. Die Autobauer schließen ihre Werke und die Airlines fliegen ohne Passagiere, um ihre Slots an den Flughäfen zu behalten. Das bedeutet: Die Maßnahmen müssen konkreter werden und die Politik muss lenkend eingreifen.

## Transparenz, Entschiedenheit und europäische Kooperation

Die Ausgangslage für die deutsche Politik ist günstig: Die Notenbanken haben mit starken Maßnahmen ihren Teil zur Krisenbewältigung beigetragen, auch fiskalpolitisch wurde schnell und gekonnt agiert, um die Unsicherheit zu begren-

1 Die frühe fiskalpolitische Reaktion in Deutschland wird in Bofinger et al. (2020) dargestellt und diskutiert.

2 Für den Einsatz des OMT-Programms unter sehr milder Konditionalität hat sich zuletzt Olivier Blanchard (2020) ausgesprochen.

3 Ebenfalls in Baldwin und Weder di Mauro (2020) spricht sich Jordi Gali unter dem Titel „Helicopter Money: The time is now“ für den tatsächlichen Einsatz von Helikoptergeld aus, d. h. einem direkt monetär finanzierten und nicht an Rückzahlung gebundenen fiskalischen Transfer.

zen. Deutschland hat mit den selbst auch längerfristig negativen Zinsen auf Anleihen derzeit beste Voraussetzungen, um nachhaltige Verbesserungen durch Investitionen herbeizuführen. Dazu verfügt die Bundesrepublik über eine solide Arbeitslosenversicherung und ein sehr gutes Gesundheitssystem. Jetzt ist eine Strategie für die Abläufe auch abseits der Welt des Geldes von Nöten. Um Unmut zu verhindern, muss klar werden, wie lange das öffentliche Leben stillstehen soll.

Ein konzertiertes europäisches Vorgehen könnte brachliegende Ressourcen dort nutzbar machen, wo sie am dringendsten gebraucht werden. So sind mit dem Stillstand des zivilen Flugverkehrs große Kapazitäten frei geworden, um Helfende oder Materialien schnell dort einzusetzen, wo sie gebraucht werden. Personen aus besonders betroffenen Industrien könnten durch Schulungen befähigt werden, in der Pflege einen guten Dienst zu erweisen und ihr Arbeitseinkommen zu sichern. Die kreativen Berufe könnten Universitäten bei der Digitalisierung und dem Ausbau ihres Onlineangebots unterstützen. Es fehlt nicht an Aufgaben oder Möglichkeiten. Die Digitalisierung sollte vorangebracht werden. Ebenso der Ausbau einer flächendeckenden Abdeckung durch das 5G-Internet. Die Förderung der digitalen Affinität ist besonders gut mit der Situation vereinbar. Unternehmen könnten dabei unterstützt werden, ihre Wertschöpfungsketten resilienter und nachhaltiger zu machen.

Eine zweckgebundene europäische Anleihe für Gesundheitsausgaben würde nationale Haushalte entlasten und hätte als sicherer Hafen gleichzeitig das Potenzial, die Finanzmärkte zu beruhigen (European Commission, 2020). Gleichzeitig könnte sie den humanitären Zusammenhalt in Europa stärken. Für alle, die das nicht überzeugt, sei gesagt: Mehr als die Hälfte der Wirtschaftsleistung Deutschlands wird im Ausland erwirtschaftet. Lahmt Europa, ist die deutsche Volkswirtschaft immer mitbetroffen.

In Italien wurde bereits begonnen, Open-Source-Beatmungsgeräte von Zuhause aus mit dem 3D-Drucker herzustellen und so Leben zu retten. Es wird sicher dauern, bis wir das alle können. Wichtig ist aber, dass sich eine agile Denkweise durchsetzt, um mit den neuen Gegebenheiten kreativ umzugehen. Auch für Gesundheitsversorgung gilt: ein effizienter Umgang mit Ressourcen kann dazu beitragen, die Probleme unserer Zeit zu lösen.

**Title:** ***Fix the Supply Side! Demand Stimulus Will Not Cure What Ails***

**JEL Classification:** *E50, E60, I10*
